# Area-based disparities in non-small-cell lung cancer survival

**DOI:** 10.2340/1651-226X.2024.27507

**Published:** 2024-04-09

**Authors:** Nelly-Maria Paakkola, Antti Jekunen, Eero Sihvo, Mikael Johansson, Heidi Andersén

**Affiliations:** a Cancer Clinic, Vaasa Central Hospital, Vaasa, Finland; b School of Medical Sciences, Faculty of Medicine and Health, Örebro University, Örebro, Sweden; c Oncology Department, University of Turku, Turku, Finland; d Department of Surgery, Central Hospital of Central Finland, Jyväskylä, Finland; e Department of Radiation Sciences, Oncology, Umeå University, Umeå, Sweden; f Faculty of Medicine and Health Technology, Tampere University, Tampere, Finland

**Keywords:** Lung cancer, disparity, rural and urban areas, non-small cell lung cancer

## Abstract

**Background:**

In the Nordic countries, universal healthcare access has been effective in reducing socioeconomic disparities in non-small-cell lung cancer (NSCLC) management. However, other factors, such as proximity to healthcare facilities, may still affect access to care. This study aimed at investigating the influence of residential area on NSCLC survival.

**Methods:**

This population-based study utilized hospital records to identify NSCLC patients who underwent their initial treatment at Vaasa Central Hospital between January 1, 2016, and December 31, 2020. Patients were categorized based on their postal codes into urban areas (≤50 km from the hospital) and rural areas (>50 km from the hospital). Survival rates between these two groups were compared using Cox regression analysis.

**Results:**

A total of 321 patients were included in the study. Patients residing in rural areas (*n* = 104) exhibited poorer 12-month survival rates compared to their urban counterparts (*n* = 217) (unadjusted Hazard Ratio [HR]: 1.38; 95% Confidence Interval [CI]: 1.01–1.89; *p* = 0.042). After adjusting for factors such as performance status, frailty, and stage at diagnosis in a multivariate Cox regression model, the adjusted HR increased to 1.47 (95% CI: 1.07–2.01; *p* = 0.017) for patients living in rural areas compared to those in urban areas.

**Interpretation:**

The study findings indicate that the distance to the hospital is associated with increased lung cancer mortality. This suggests that geographical proximity may play a crucial role in the disparities observed in NSCLC survival rates. Addressing these disparities should involve strategies aimed at improving healthcare accessibility, particularly for patients residing in rural areas, to enhance NSCLC outcomes and reduce mortality.

## Background

With modern treatment options, lung cancer survival has increased in the Nordic countries. In Finland compared to other Nordic countries, this improvement has, however, been slower. Most recent national 5-year survivals by gender in Finland and Norway were 16.4% versus 26.6% in males and 25.5% versus 33.2% in females, respectively [1]. In the study area, Ostrobothnia with a lung cancer incidence of 57.84 per 100,000 in 2020, the mortality incidence ratio was above the Finnish median [2]. The underlying causes of these survival disparities remain unclear.

In theory, the governmentally funded healthcare systems employed in the Nordic countries should serve to mitigate socioeconomic disparities in cancer management. Therefore, modern treatment options should be uniformly accessible for lung cancer patients. In practice, variables such as proximity to healthcare facilities have a potential effect on treatment decisions and constrain healthcare accessibility. Travelling time and distance can significantly hinder patient adherence to treatments requiring high compliance due to repeated visits such as multi-fraction radiation therapy or intensive chemoradiotherapy regimens.

Previous lung cancer studies have indicated mixed outcomes between survival and the area of residence [3–9]. The place of residence in the United States [3, 4] and in France [5] has been associated with overall lung cancer survival, whereas an Australian [6] and a Polish study [7] found no association. Few Nordic or Finnish studies on the effect of distance on lung cancer survival or area-based disparities in cancer management exist. The use of palliative radiotherapy has been shown to be decreased with long travelling distances in Norway [8]. A recent Swedish study found no association between travelling time and survival for patients with colorectal cancer [9].

Given the unique healthcare systems prevalent in the Nordic region, earlier studies on how travel distance affects lung cancer survival might not apply here. Therefore, there is a need for studies specifically examining disparities based on geographical areas within this Nordic healthcare context. Our objective was to evaluate the influence of proximity to the hospital on the survival rates of individuals with non-small-cell lung cancer (NSCLC) in the region of Ostrobothnia, Finland.

## Material and methods

This retrospective study consisted of all patients diagnosed with the ICD-10 diagnosis code of C34 for NSCLC who had their first treatment at the Vaasa Central Hospital in Finland between January 1, 2016 and December 31, 2020. Vaasa Central Hospital offers extensive diagnostic services, including Endobronchial Ultrasound (EBUS) and Fluorodeoxyglucose Positron Emission Tomography (FDG PET CT), alongside oncologic treatments that encompass radiotherapy with curative intent. However, lung cancer surgeries are performed at a different facility.

The variables extracted from the patient records were age, biological sex, World Health Organization Performance Status (PS), body mass index (BMI), forced expiratory volume (FEV1), and smoking history. Patients were defined as never-smokers if they had smoked less than one pack during their lifetime, ex-smokers if they had not smoked within the previous year, and current smokers if they had smoked within the previous year. Patient fitness was assessed using the PS [10] and the Clinical Frailty Scale (CFS) [11], the latter retrospectively estimated from patient records. PS was categorized into two groups, 0–2 and 3–4, based on clinical rationale. With PS 0–2, according to the Finnish guidelines for NSCLC, patients were medically fit for a treatment [12]. Best supportive care should be offered to PS 3–4 patients. CFS is a scale considered highly predictive of mortality and correlates with other frailty scales [13]. The patients were categorized according to their CFS score into Robust (1–2), Pre-Frail (3–4), and Frail (≥5). Charlson Comorbidity Index (CCI) [14] was used to assess comorbidities, which were categorized into four different categories: 0, 1–2, 3–5, and 5 or more. Information on cancer characteristics extracted were stage according to the 8th edition of Tumor, Node, Metastasis-staging [15], and tumor histology. Guideline adherence considers first-line treatment for the stage of the disease and patients PS [16, 17].

A system assessing neighborhood affluence was created based on the average income, unemployment level, and number of people with tertiary education in the area using database by Statistics Finland. The areas were scored from 0 to 6 according to predefined criteria and categorized as impoverished (0–2), average (3–4), or affluent (5–6) neighborhoods (Supplementary Table 1). Individuals’ occupations and their skill levels according to the International Standard Classification of Occupations (ISCO-08) were utilized as indirect indicators of their socioeconomic status. The main variable considered was the distance to the hospital. The postal codes for each patient were collected, and the distance to the hospital was recorded based on the postal code. Rural area was defined as >50 km straight-line-distance from the hospital, as this was considered an hour’s transport time to the hospital. The urban area was defined as ≤50 km distance from the hospital (Supplement Figure 1). The distance cut-off was chosen based on prior literature and practical considerations related to patient travel time and access to healthcare services.

The primary outcome assessed was overall survival (OS) calculated from the first day of treatment or the decision of best supportive care to the date of death or date of last follow-up. The follow-up ended on March 2, 2022.

### Statistical analysis

Categorical variables are presented as frequencies and percentages. Differences in categorical variables were compared across urban and rural categories by using Chi-Square tests if the sample size in all cells was ≥5 or Fisher’s exact tests if the sample size in different categories was <5. Continuous variables are presented as median and interquartile range (IQR). Differences in continuous variables were compared across urban and rural groups by using the Mann–Whitney U test. All statistical tests were two-sided. The test was considered statistically significant if *p* < 0.05.

Total median OS and OS for urban and rural patients were calculated using the Kaplan Meier estimate at the data cut-point on the 2nd of March 2022. A univariate analysis for 1-year OS was conducted, and the groups were compared using the Chi-Square test. Variables that were correlated with survival in the univariate analysis with a *p* < 0.05 were included in the multivariate analysis. A multivariate Cox regression model was used to calculate the hazard ratios (HR) with 95% confidence intervals (CI) for the relative risk of death of all causes.

## Results

**Figure 1:**
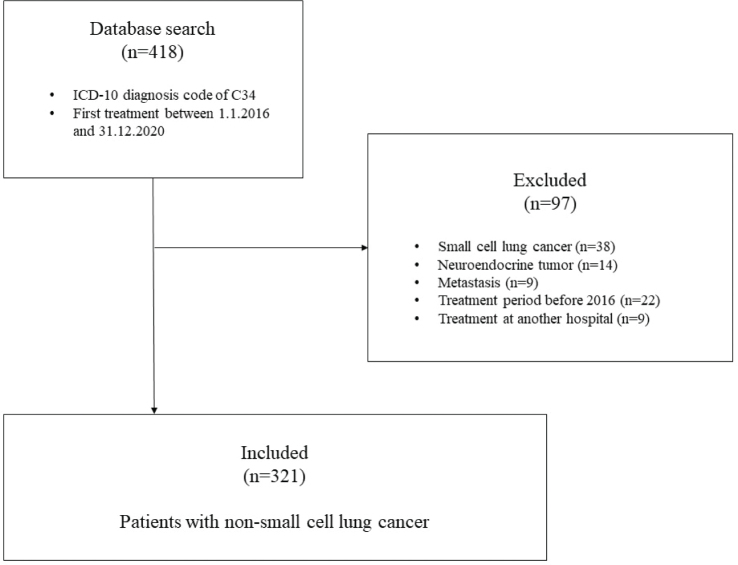
Flow chart of the study.

The study encompassed a total of 321 patients diagnosed with NSCLC, of which 217 (67.6%) were urban residents and 104 (32.4%) rural residents (Figure 1). Demographic and clinical characteristics, including age, sex, BMI, FEV1, PS, CFS, CCI, histology, or TNM stage, were statistically analogous between urban and rural groups (Table 1). The rural patients had a lower median pack-years of smoking compared to their urban counterparts (30 vs. 40 pack-years, *p* = 0.011). Furthermore, a marked disparity in socioeconomic status, as inferred from neighborhood affluence, was observed between the rural and urban populations. The proportion of patients residing in affluent areas was higher among urban residents, 24.4% compared to 10.6% among rural residents. Conversely, the rural population exhibited a greater percentage of individuals from less-affluent backgrounds.

**Table 1:** Patient demographics in urban and rural non-small-cell lung cancer patients.

	Total *N* = 321	Urban (≤50 km) *n* = 217 (67.6)	Rural (>50 km) *n* = 104 (32.4)	*p*
**Sex**				0.858
Male	209 (65.1)	142 (65.4)	67 (64.4)	
Female	112 (34.9)	75 (34.6)	37 (35.6)	
**Median age in years (IQR)**	73 (67, 78)	73 (67, 78)	73 (69, 78)	0.534
**Median BMI (IQR)**	25.7 (22.9, 28.0)	25.9 (23.0, 28.2)	24.6 (22.7, 27.6)	0.255
**Smoking history**				0.112
Current	127 (39.6)	91 (41.9)	36 (34.6)	
Former	150 (46.7)	102 (47.0)	48 (46.2)	
Never	44 (13.7)	24 (11.1)	20 (19.2)	
**Median pack-years (IQR)**	40 (13, 50)	40 (20, 50)	30 (2, 49)	0.011
**Performance status (PS)**				0.389
0–2	250 (77.9)	172 (79.3)	78 (75.0)	
3–4	71 (22.1)	45 (20.7)	26 (25.0)	
**Clinical Frailty Scale**				0.824
Robust (1–2)	74 (23.1)	50 (23.0)	24 (23.1)	
Pre-Frail (3–4)	112 (34.9)	78 (35.9)	34 (32.7)	
Frail (≥5)	135 (42.1)	89 (41.0)	46 (44.2)	
**TNM (8^th^ edition) stage**				0.925
I-II	74 (23.1)	50 (23.0)	24 (23.1)	
III	64 (19.9)	42 (19.4)	22 (21.2)	
IV	183 (57.0)	125 (57.6)	58 (55.8)	
**Disseminated metastatic (IVb)**	89 (48.9)	57 (45.6)	32 (56.1)	0.187
**Histologic type**				0.329
Adenocarcinoma	189 (58.9)	123 (56.7)	66 (63.5)	
Squamous	89 (27.7)	61 (28.1)	28 (26.9)	
Other & unknown	43 (13.4)	33 (15.2)	10 (9.6)	
**Genetic testing not done**	71 (22.1)	46 (21.2)	25 (24.0)	0.365
**Median FEV1% (IQR)**	69 (53, 85)	67 (54, 85)	76 (52, 92)	0.277
**Charlson Comorbidity Index**				0.777
0	87 (27.1)	62 (28.6)	25 (24.0)	
1–2	180 (56.1)	119 (54.8)	61 (58.7)	
3–5	39 (12.1)	25 (11.5)	14 (13.5)	
5 or more	15 (4.7)	11 (5.1)	4 (3.8)	
**Guideline adherence**				0.506
Limited treatment for stage and PS	82 (25.5)	53 (24.4%)	29 (27.9%)	
Adherent treatment	239 (74.5)	164 (75.6%)	75 (72.1%)	
**First line treatment**				0.144
Surgery	56 (17.4)	37 (17.1)	19 (18.3)	
Curative intent radiotherapy	44 (13.7)	29 (13.4)	15 (14.4)	
Systemic therapy	104 (32.4)	76 (35.0)	28 (26.9)	
Palliative radiotherapy	60 (18.7)	33 (15.2)	27 (26.0)	
Best supportive care	57 (17.8)	42 (19.4)	15 (14.4)	
**Affluence of the area**				<0.001
Affluent	64 (19.9)	53 (24.4)	11 (10.6)	
Average	117 (36.4)	40 (18.4)	77 (74.0)	
Impoverished	140 (43.6)	124 (57.1)	16 (15.4)	
**ISCO skill level**				0.383
1–2, less than 12 years of education	212 (77.7)	144 (76.2)	68 (81.0)	
3–4, more than 12 years of education	61 (22.3)	45 (23.8)	16 (19.0)	
**Median distance to hospital in km (IQR)**	20 (4, 69)	7 (3, 23)	86 (69, 88)	<0.001

Regarding the cancer stage, there was no significant difference in the distribution of stages between rural and urban dwellers, but a shift towards more disseminated disease was observed among rural than urban residents. There was a noted tendency for less frequent application of systemic therapy in rural patients compared to their urban counterparts. In the study, 71 (22.1%) participants did not undergo genetic testing, with 46 (21.2%) from urban areas and 25 (24.0%) from rural areas, showing no significant difference in testing rates (*p* = 0.365). Treatment adherence to guidelines was observed in 239 (74.5%) of cases, with urban residents at 75.6% and rural at 72.1%, indicating similar adherence levels across locations (*p* = 0.506) (Table 1).

### Survival

**Figure 2:**
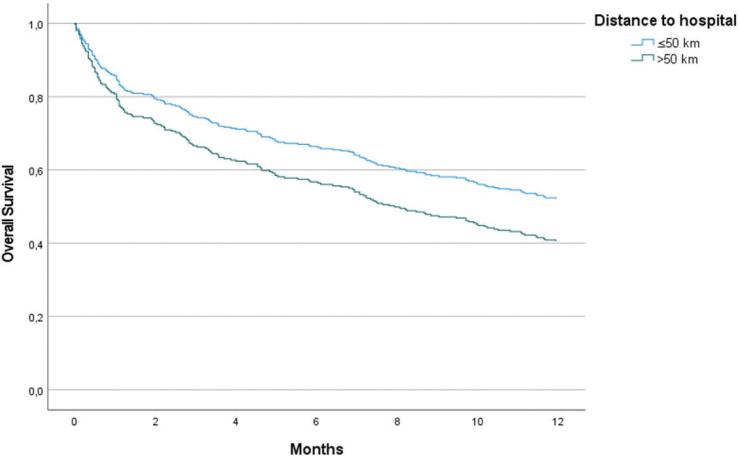
Cox Proportional-Hazard Model for Overall Survival (OS) in NSCLC Patients, Adjusted for Performance Status (PS), Clinical Frailty Scale (CFS), and Stage at Diagnosis. Hazard Ratio (HR): 1.47 (95% Confidence Interval [CI]: 1.07–2.01, *p* = 0.017).

Overall median survival in the study group was 11.2 months (95% CI: 8.7–13.7 months) (Table 2). When differentiating by residency, urban patients had a median survival of 12.9 months (95% CI: 9.6–16.2 months), and rural patients had a median survival of 8.8 months (95% CI: 5.9–11.7 months). The 1-year survival rate was observed to be higher in urban patients at 52.5% as compared to 39.4% in rural patients (*p* = 0.028). In the univariate analysis, factors having a significant effect on 1-year survival were PS, stage, and CFS (Table 3). After adjustment for these factors in a multivariate Cox regression model, the adjusted HR for 1-year survival was 1.47 (95% CI: 1.07–2.01, *p* = 0.017) for patients living in rural compared to urban areas. In the adjusted model, in addition to accessibility, PS, CFS, and stage remained statistically significant for survival (Table 4). The Cox proportional-hazards survival graph is presented in Figure 2.

**Table 2:** Overall survival raw numbers year 0–3.

Urban (≤50 km)	3 months	1 year	2 year	3 year
		*n* (%)	*n* (%)	*n* (%)
Patients at risk	161 (74.2)	114 (52.5)	67 (30.9)	38 (17.5)
Number of deaths	56 (25.8)	103 (47.5)	132 (60.8)	150 (69.1)
Endpoint not reached	0 (0.0)	0 (0.0)	18 (8.3)	29 (13.4)
**Rural (>50 km)**				
Patients at risk	70 (67.3)	41 (39.4)	24 (23.1)	14 (13.5)
Number of deaths	34 (32.7)	63 (60.6)	74 (71.2)	79 (76.0)
Endpoint not reached	0 (0.0)	0	6 (5.8)	11 (10.6)

**Table 3:** Analysis of overall survival 1-year.

Variables	Overall survival 1-year		
	*N*	%	*P*
**Sex**			0.800
Male	102	48.8	
Female	53	47.3	
**Age**			0.438
≤73	87	50.3	
>73	68	45.9	
**BMI**			0.689
<18.5	5	54.5	
18.5–30	108	56.3	
≥30	20	51.3	
**Smoking status**			0.510
Current smoker	57	44.9	
Ex-smoker	74	49.3	
Never smoker	24	54.5	
**Pack-years**			0.565
<40	75	50.0	
≥40	80	46.8	
**WHO performance status**			<0.001
0–2	*147*	*58.8*	
3–4	*8*	*11.3*	
**Clinical Frailty Scale**			<0.001
Robust (1–2)	*68*	*91.9*	
Pre-Frail (3–4)	*60*	*53.6*	
Frail (≥5)	*27*	*20.0*	
**Stage at diagnosis**			<0.001
I-II	*67*	*90.5*	
III	*37*	*57.8*	
IV	*51*	*27.9*	
**Disseminated metastatic (IVa vs. IVb)**	16	18.0	0.005
**Histological type**			0.274
Adenocarcinoma	96	50.8	
Squamous	43	48.3	
Other & unknown	16	37.2	
**FEV1**			0.438
<50%	18	60.0	
≥50%	95	67.4	
**Charlson Comorbidity Index**			0.490
0	40	46.0	
1–2	92	51.1	
3–4	15	38.5	
5 or more	8	53.3	
**Guideline adherence**			0.039
Limited treatment for stage and PS	37	39.4	
Adherent treatment	118	52.0	
**First line treatment**			<0.001
Surgery	55	98.2	
Curative intent radiotherapy	29	65.9	
Systemic therapy	54	51.9	
Palliative radiotherapy	12	20	
Best supportive care	5	8.8	
**Affluence of the area**			0.347
Affluent	28	43.8	
Average	53	45.3	
Impoverished	74	52.9	
**ISCO skill level**			0.509
1–2, less than 12 years of education	108	50.9	
3–4, more than 12 years of education	34	55.7	
**Distance to hospital**			0.028
Urban (<50 km)	114	52.5	
Rural (≥50 km)	41	39.4	

**Table 4:** Multivariate Cox regression survival analysis.

Variables	Overall survival 1-year		
	Hazard ratio	95% confidence interval	*P*
**Distance to hospital**			
Urban (<50 km) ref.	1.00		
Rural (≥50 km)	1.47	1.07–2.01	0.017
**Performance Status**			
0–2 ref.	1.00		
3–4	2.24	1.55–3.25	<0.001
**Clinical Frailty Scale**			
Robust 1–2 ref.	1.00		
Pre-Frail 3–4	4.74	2.02–11.12	<0.001
Frail 5 ≥5	8.48	3.59–20.06	<0.001
**Stage**			
I–II ref.	1.00		
III	4.46	1.94–10.27	<0.001
IV	8.01	3.71–17.28	<0.001

## Discussion

Our study suggests that proximity to the hospital significantly influenced the survival outcomes of lung cancer patients. Patients from urban areas in Ostrobothnia, Finland, exhibited a survival advantage over their rural counterparts. In line with findings from prior studies, other key factors associated with survival were CFS, PS, and stage.

Research consistently demonstrates that individuals residing farther from healthcare facilities, particularly those offering specialized cancer care, often encounter geographical disparities in accessing timely diagnosis and treatment for lung cancer [18, 19]. Rural areas, in particular, confront formidable challenges related to healthcare access, including extended travel times and a scarcity of healthcare facilities and access to screening [19]. Consequently, lung cancer mortality rates tend to be elevated in rural regions, partially attributed to these access-related impediments. Consistent with our study, research conducted in the United States [3, 4] and France [5] has also identified an unfavorable correlation between rural residence and overall survival in lung cancer patients. Conversely, studies conducted in Australia [6] and Poland [7] found no such association.

This study found no difference in stage at diagnosis between urban and rural residents. Previous studies show that rural residents get diagnosed at a later stage [20, 21], and may therefore have a cancer survival disadvantage [22]. The underlying reasons for the survival disadvantages observed among rural residents in this study remain uncertain. Nevertheless, it is notable that rural patients exhibited a consistent pattern: a higher prevalence of metastatic disease, an increased requirement for palliative radiotherapy, and a lower utilization of systemic therapy when compared to their urban counterparts. The cumulative impact of these factors may provide an explanation for the observed differences in survival, particularly in advanced stages.

Although the Finnish healthcare system is founded on the principle of equal access for all residents, the practical accessibility of care may fluctuate depending on one’s place of residence. Primary care functions as a gatekeeper, typically necessitating a referral from a general practitioner for specialist care [23]. A total of 56.1% of Ostrobothnians have access to a primary care centre within a 30-min travel time via public transport [24]. It’s plausible that rural patients might be less inclined to seek medical attention or their access to primary care is limited.

This study found no difference in the rate of curative aimed therapy or guideline adherence between urban and rural residents. The most intricate aspects of cancer management are centralized, with the nearest University hospital being over a 3-h drive from Vaasa Central Hospital [25]. Notably, lung cancer surgery is concentrated in a high-volume centre, ensuring that all patients receive high-quality, guideline-adherent surgical interventions. In a registry-based study from the United States, poorer survival for rural patients compared to urban patients was reported as they were less likely to be treated at high-volume centers [26]. Unlike our study, Ray et al. reported limited guideline concordance as a source of rural–urban survival disparity [27]. Previously reported rural–urban disparities in genomic testing [28] were not present in our study.

In addition, the extended travel distances may result in increased treatment non-compliance, potentially attributed to factors like prolonged travel times, lack of accessible transportation, and financial constraints [29, 30]. Socioeconomic factors, encompassing income and insurance status, can exert an additional influence on lung cancer mortality [31]. To address these challenges, Finland has implemented several strategies aimed at mitigating the impact of distance on healthcare accessibility. Notably, the Finnish National Health Insurance offers reimbursements for travel expenses incurred during visits to healthcare providers. Additionally, telemedicine and mobile healthcare units represent innovative approaches employed in many rural areas to overcome distance-related access obstacles. These strategies collectively contribute to reducing the impact of transportation barriers and enhancing the accessibility of healthcare services, especially in remote regions.

### Strengths and limitations

The investigation is a population-based retrospective study, with the geographic scope confined to a specific region, which enhances the homogeneity of the population sample. The single centre study design and its relatively small sample size were the primary limitations. Verification by the Finnish Cancer Registry supports the reliability of the data and confirms minimal inclusion bias, adding to the study´s credibility. Mortality and incidence rates, slightly higher than the Finnish median, enable the consideration of the findings as potentially extrapolative to the broader Finnish setting, albeit with caution. CFS was estimated retrospectively. Occupation and skill level were used as a proxy of socioeconomic status, as data on income or education were not available.

The sample size may not be sufficient for subgroup analyses, which could limit the ability to detect statistically significant differences or to conduct a granular assessment of the data. The absence of lung cancer screening programs in Finland at the time of the study means that potential early detection biases are not present, which could differ in settings where such programs are active. The study´s findings are subject to the limitation of not accounting for variations in access to healthcare facilities, which is a known contributor to disparities in mortality rates. Recent structural changes in hospital networks and cancer centre distributions are not reflected in the study but are recognized as factors that could exacerbate inequalities in patient outcomes. Overall, the study presents valuable insights on challenges associated with travel to healthcare facilities while also identifying areas that require careful consideration when interpreting the findings and in the context of healthcare policy implications.

## Conclusion

This study found an association between proximity to the hospital and the 1-year OS of NSCLC patients. Further research is warranted to delve into the underlying causes of these survival disparities with finer granularity. To mitigate these disparities, there is a clear need to develop strategies aimed at enhancing healthcare accessibility, particularly for residents of geographically remote areas. The improvement of lung cancer care pathways is essential to ensure more equitable access to healthcare for all individuals affected by NSCLC.

## Supplementary Material

Area-based disparities in non-small-cell lung cancer survival

## Data sharing statement

According to the ethical permission and data-protection laws of Finland, single-person data cannot be made available.
