# Supplementary material for: Area-based disparities in non-small-cell lung cancer survival
Source: Acta Oncol. 2024 Apr 9;63:27507. doi: 10.2340/1651-226X.2024.27507 (PMC11332544; doi:10.2340/1651-226X.2024.27507)

Supplementary material has been published as submitted. It has not been copyedited or typeset by Acta Oncologica.

Supplement

Area-based disparities in non-small-cell lung cancer survival

Nelly-Maria Paakkola, Antti Jekunen, Eero Sihvo, Mikael Johansson, Heidi Andersén

Supplement Table 1. A novel system assessing neighbourhood affluence was created based on the average income, unemployment level, and number of people with tertiary education in the area. The areas were scored from 0-6 according to predefined criteria and categorized as impoverished (0-2), average (3-4) or affluent (5-6) neighbourhoods. The quartiles were based on 2022 Statistics Finland data.

**A system assessing the neighborhood affluence, based on quartiles (Q) of the area 2022**			
	Q1	Q2-Q3	Q4
Criteria 1:<br>**Yearly income**	≤22,706€	22,707 – 25,833€	≥25,835€
Criteria 2:<br>**Tertiary education**	≤15.8%	15.9 - 29.8%	≥29.9%
Criteria 3:<br>**Unemployment rate**	≥8.7%	4.7 – 8.6%	≤4.6%
Score	0 points for each category	1 point for each category	2 points for each category
**Neighborhood affluence criteria**			
	Total score	Minimum criteria	
**Inaffluent area**	0-2	-	
**Average area**	3-4	≥1 point for each criteria	
**Affluent area**	5-6	≥2 points at least from 2 criteria	

Supplement Figure 1. 50 km distance from Vaasa Central Hospital

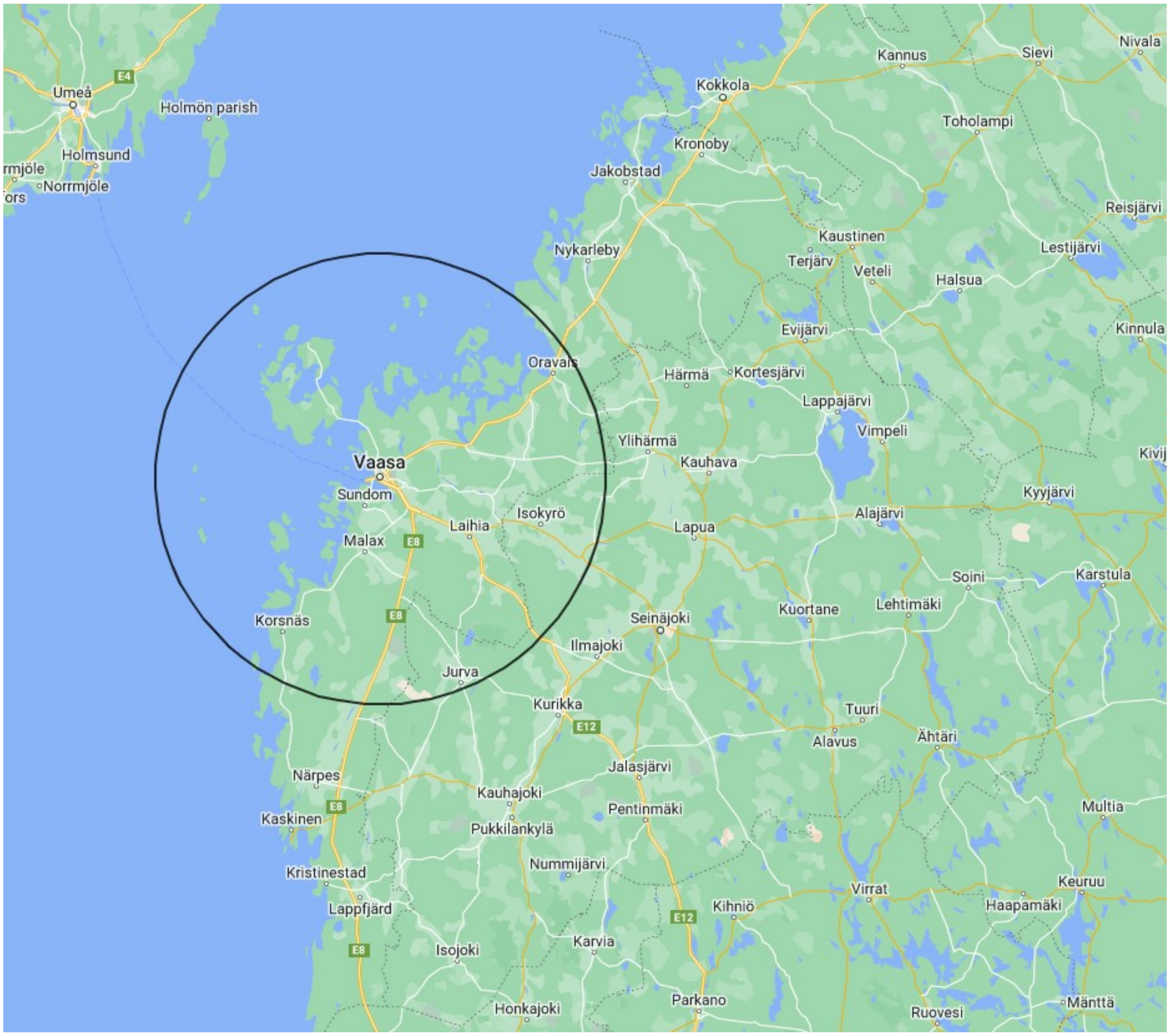

Supplement: Area-based disparities in non-small-cell lung cancer survival [file AO-63-27507-s1.pdf]
